# Heterostructures Made of Upconversion Nanoparticles and Metal–Organic Frameworks for Biomedical Applications

**DOI:** 10.1002/advs.202103911

**Published:** 2021-11-17

**Authors:** Qing Liu, Bo Wu, Mengyuan Li, Yuanyu Huang, Lele Li

**Affiliations:** ^1^ School of Life Science Institute of Engineering Medicine Key Laboratory of Molecular Medicine and Biotherapy Beijing Institute of Technology Beijing 100081 China; ^2^ CAS Key Laboratory for Biomedical Effects of Nanomaterials and Nanosafety and CAS Center for Excellence in Nanoscience National Center for Nanoscience and Technology Beijing 100190 China; ^3^ School of Chemistry and Biological Engineering University of Science and Technology Beijing Beijing 100083 China

**Keywords:** bioimaging and biosensing, heterostructures, metal‐organic frameworks, photodynamic therapy, upconversion nanoparticles

## Abstract

Heterostructure nanoparticles (NPs), constructed by two single‐component NPs with distinct nature and multifunctional properties, have attracted intensive interest in the past few years. Among them, heterostructures made of upconversion NPs (UCNPs) and metal–organic frameworks (MOFs) can not only integrate the advantageous characteristics (e.g., porosity, structural regularity) of MOFs with unique upconverted optical features of UCNPs, but also induce cooperative properties not observed either for single component due to their special optical or electronic communications. Recently, diverse UCNP‐MOF heterostructures are designed and synthesized via different strategies and have demonstrated appealing potential for applications in biosensing and imaging, drug delivery, and photodynamic therapy (PDT). In this review, the synthesis strategies of UCNP‐MOF heterostructures are first summarized, then the authors focus mainly on discussion of their biomedical applications, particularly as PDT agents for cancer treatment. Finally, the authors briefly outlook the current challenges and future perspectives of UCNP‐MOF hybrid nanocomposites. The authors believe that this review will provide comprehensive understanding and inspirations toward recent advances of UCNP‐MOF heterostructures.

## Graphical abstract

Heterostructures consisting of upconversion nanoparticles and metal‐organic frameworks represent powerful nanoplatforms for diverse biomedical applications. This review summarizes the key strategies for the synthesis of such structures and their potential applications in biosensing and imaging, drug delivery, and photodynamic therapy.

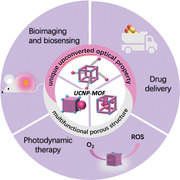

## 1 Introduction

Metal–organic frameworks (MOFs) are 2D or 3D porous crystalline materials formed by self‐assembly of metal ions or clusters with multitopic organic linkers.^[^ ^1^ ^]^ In the past decade, varieties of MOFs with tunable structures and well‐defined pores have been designed and synthesized through combinations of different building blocks.^[^ ^2^ ^]^ The intrinsic structural characteristics of MOFs, such as regular pores, controllable pore size, and large surface area, enable them capture sufficient guest molecules and achieve high loading efficiency.^[^ ^3^ ^]^ Benefiting from their unique physical and chemical properties, MOFs have been extensively explored in the fields of gas storage and separation,^[^ ^4^ ^]^ catalysis,^[^ ^5^ ^]^ drug delivery,^[^ ^6^ ^]^ chemical sensing, and disease diagnosis.^[^ ^7^ ^]^ Development of post‐modification strategy has imparted MOFs with additional chemical functionalities^[^ ^8^ ^]^ and largely boosted their application potential, especially in biomedical areas.^[^ ^9^ ^]^ Recently, much efforts have been devoted to integrate MOFs with other nanomaterials, such as metal NPs,^[^ ^10^, ^11^, ^12^ ^]^ carbon nanotube,^[^ ^13^ ^]^ and even another MOFs,^[^ ^14^ ^]^ to construct multifunctional heterostructures. Such structures not only retain the properties of MOFs but also bestow with other attractive functions from their counterparts, therefore achieving synergistic effect for enhanced performance.^[^ ^15^, ^16^ ^]^

Upconversion NPs (UCNPs) possess nonlinear anti‐Stokes properties through lanthanide ion doping.^[^ ^17^ ^]^ They can sequentially absorb two or more photons under long‐wavelength radiation (e.g., NIR) and convert them into tunable, short‐wavelength UV or visible emissions.^[^ ^18^, ^19^, ^20^ ^]^ Up to date, UCNPs have been processed by size regulation, host‐lattice modulation, core‐shell engineering, and surface ligands exchange and have been endowed with unique photochemical characteristics,^[^ ^21^ ^]^ such as narrow emission bandwidth, resistance to photobleaching, long luminescence lifetime, and relatively higher photon energy.^[^ ^22^ ^]^ Such fascinating characteristics make them promising materials to be used as biosensors,^[^ ^23^, ^24^ ^]^ imaging agents,^[^ ^25^ ^]^ and therapeutics.^[^ ^26^, ^27^, ^28^ ^]^ However,the applications of UCNPs in biomedical areas are still facing challenges due to the limited surface engineering strategies and low drug loading capacity.

**Figure 1:**
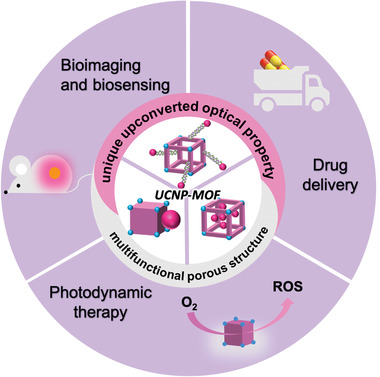
Schematic representation of UCNP‐MOF heterostructures for diverse biomedical applications.

Recently, researchers have developed several methods to construct UCNP‐MOF heterostructures to integrate the superior functionality of MOFs with the optical‐upconverting capability of UCNPs. Furthermore, such heterostructures may possess cooperative functions originating from the optical or electronic communications between UCNPs and MOFs thus overcoming the limitations of each individual material (e.g., low drug loading capacity of UCNPs, the narrow light absorption of porphyrinic MOFs in visible region). Therefore, the UCNP‐MOF heterostructures are conducive to accomplish improved performances compared to the single component, especially in biomedical applications.^[^ ^29^ ^]^ Herein, we provide a concentrated overview of recent advances in UCNP‐MOF heterostructures (**Figure** **1**). We first summarize the synthesis strategies of these functional heterostructures. Then, we mainly focus on its biomedical applications involving bioimaging and biosensing, drug delivery, and photodynamic therapy (PDT). Finally, a brief discussion on existing challenges and future perspectives are presented.

## 2 Controlled Synthesis of UCNP‐MOF Heterostructures

### 2.1 Surface Ligand‐Mediated Synthesis

A variety of UCNPs have been synthesized by using different strategies such as seed‐assisted growth^[^ ^30^ ^]^ and successive layer‐by‐layer strategy.^[^ ^31^, ^32^ ^]^ To obtain high‐quality NPs, oleic acid (OA) with a coordinating headgroup has been chosen as a capping ligand in most of the preparation processes. Such OA‐capped UCNPs cannot be dispersed in polar solvents, which are often required for the subsequent formation of UCNP‐MOF heterostructures. To solve this problem, researchers have developed ligand exchange strategies to replace OA molecules with hydrophilic ligands, such as polyvinylpyrrolidone (PVP) or molecules containing carboxyl groups. These ligands not only help UCNPs disperse easily in polar solvents but also facilitate growth of MOFs onto UCNPs.^[^ ^33^, ^34^ ^]^

As an amphiphilic polymer, PVP is the most commonly adopted ligand for fabrication of UCNP‐MOF heterostructures due to its strong coordination capability with crystal‐forming ions.^[^ ^35^ ^]^ Li group presented a facile approach to construct core‐shell UCNP@MIL‐53(Fe) NPs using PVP as the mediation ligand.^[^ ^36^ ^]^ After the synthesis of NaYF_4_:Yb,Tm UCNPs, OA was removed by diluted HCl solution and a PVP layer was sequentially coated on their surface. The pyrrole structure in PVP has strong coordination interaction with Fe^3+^ ions, thus Fe^3+^ ions could be absorbed and enriched on the nanoplates. After addition of terephthalic acid (TPA) linker, nucleation of MIL‐53(Fe) started preferably on the surface of the UCNPs rather than homogeneous nucleation in the solution, resulting in the formation of core‐shell UCNP@MOF heterostructures. Moreover, the thicknesses of MOF shell can be tuned by adjusting the concentration of raw materials for MOF synthesis.

**Figure 2:**
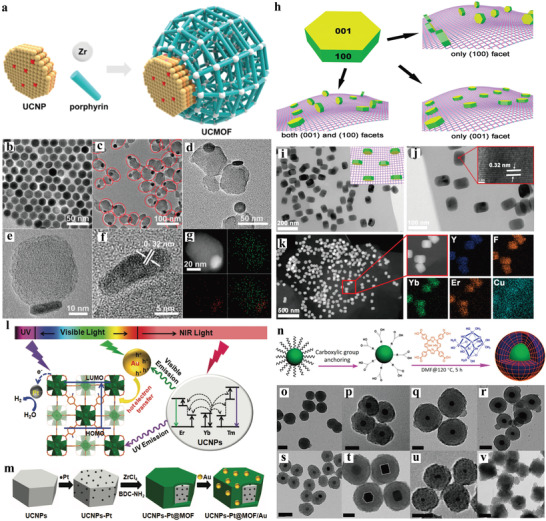
a) Schematic illustration of the synthesis of UCMOF heterodimers via the anisotropic growth of MOFs onto PVP‐modified UCNPs. b) TEM image of UCNPs. c,d) TEM and e,f) HRTEM images of UCMOFs. The red ellipses highlighted the dimers in (c). g) HAADF‐STEM image and corresponding elemental mapping images (green for Zr and red for Gd) of a single UCMOF. Reproduced with permission.^[^ ^37^ ^]^ Copyright 2017, American Chemical Society. h) Three possibilities for assembly of UCNPs onto the surface of 2DMOFs. i,j) TEM images, k) HAADF image, and EDS elemental mapping of the UCNPs/2DMOFs nanostructures. Reproduced with permission.^[^ ^38^ ^]^ Copyright 2021, Wiley‐VCH. l) The light absorption and involved energy transfer mechanism of the UCNPs‐Pt@MOF/Au composites. m) Schematic illustration of the synthesis of UCNPs‐Pt@MOF/Au composites. Reproduced with permission.^[^ ^39^ ^]^ Copyright 2018, Wiley‐VCH. n) Schematic illustration of the fabrication of core‐shell structures through anchoring carboxylic groups on the surfaces of colloidal NPs and further growth of MOFs. TEM images of o) ZrMOF, p) iron oxide@ZrMOF, q) Au‐20@ZrMOF, r) Au‐50@ZrMOF, s) UCNP‐sphere@ZrMOF, t) UCNP‐hexahedron@ZrMOF, u) Ag@ZrMOF, and v) UiO‐66@ZrMOF. Reproduced with permission.^[^ ^42^ ^]^ Copyright 2019, American Chemical Society.

Recently, we reported the first NIR light‐harvesting, asymmetric UCNP‐MOF heterodimers (UCMOFs) by selective anisotropic growth of Zr‐based porphyrinic MOFs onto UCNPs (**Figure** **2**a).^[^ ^37^ ^]^ By mixing the MOF precursors with PVP‐coated core‐shell UCNPs (NaGdF_4_:Yb,Er@NaGdF_4_), UCMOFs with anisotropically presented MOF domain on the top surfaces of UCNPs were obtained (Figure 2b–g). According to the statistical analyses, ~81% yield of heterodimers was achieved under the optimal condition. Exploration of synthesis condition suggested that the high yield of heterodimers was relevant to the optimal amount of UCNP seeds. Whether a decrease or increase concentration of UCNP seeds would result in an increased population of isolated MOF NPs or MOF NP with multiple UCNPs on its surface. In terms of PVP ligands, when the UCNPs were not functionalized with PVP, self‐nucleation of MOF NPs significantly occurred and no heterodimers were formed. Furthermore, theoretical calculations by density functional theory suggested that PVP preferentially binds to (001) facet at the top faces rather than on the (100) facet at side faces of UCNPs. All these results indicated that PVP ligands indeed exerted the crucial effect for UCNPs stabilization and nucleation of MOF domain on UCNPs. The different binding affinity of each crystal facet of seed UCNPs to the first atomic layer of secondary material is a key for the anisotropic growth of heterodimers.

Most recently, Yuan et al. reported the synthesis of UCNP/MOF heterostructures by PVP‐mediated assembly of anisotropic UCNPs with2DMOFs (Figure 2h).^[^ ^38^ ^]^ In this work, UCNPs were attached on 2DMOFs via the (100) facets, as evidenced by TEM and high‐resolution TEM images (Figure 2i–k). Increasing PVP concentration for coating UCNPs, UCNPs were randomly assembled on 2DMOFs without facet selectivity. More complicated core‐shell UCNPs‐Pt@MOF/Au nanocomposites were fabricated by Jiang et al. (Figure 2l,m). PVP‐modified UCNPs facilitated the deposition of Pt NPs and further growth of the MOF shell, followed by attachment of Au NPs on the MOF shell.^[^ ^39^ ^]^ Apart from PVP, other molecules are also suitable to mediate the growth of UCNP‐MOF heterostructures, such as citrate acid^[^ ^40^ ^]^ and poly(ethylenimine).^[^ ^41^ ^]^

Tan et al. demonstrated that carboxylic acid groups anchored on the surface of inorganic NPs through ligand exchange could allow for MOF growth (Figure 2n).^[^ ^42^ ^]^ By adding the 3,4‐hydroxycinnamic acid modified UCNPs to a MOF precursor solution, uniform core‐shell UCNP@MOF heterostructures could be obtained after incubation at 120 °C, as indicated by TEM images. It revealed that the formation of the heterostructures was a chelation‐driven coordination process. This strategy has also been validated to be applicable for a variety of NPs with different sizes and shapes (Figure 2o–v). Results showed that a dual fluorescence resonance energy transfer (FRET) occurred in UCNP@ZrMOF, which induced singlet oxygen generation under NIR irradiation. The Wang group reported another example for the synthesis of such heterostructures with polyacrylic acid (PAA)‐modified UCNPs.^[^ ^43^ ^]^ The (Cu_3_(BTC)_2_) MOFs were deposited on the surface of PAA‐UCNPs to form UCNP/MOF NPs, which could be used as substrate for molecularly imprinted polymers for protein sensing. These work indicate that the surface ligand‐mediated strategy is a feasible approach to construct UCNP‐MOF heterostructures with controlled geometry and property.

### 2.2 Electrostatic Interaction‐Driven Synthesis

**Figure 3:**
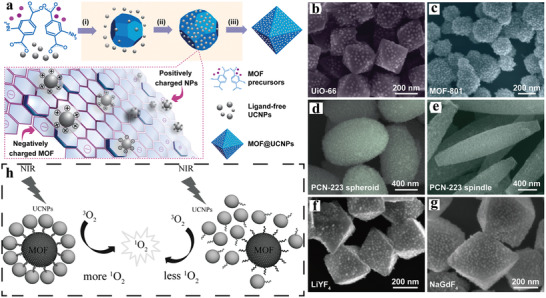
a) Schematic illustration of the synthesis of UCNP‐MOF nanocomposites via the growth of MOFs in the presence of ligand‐free UCNPs. (i) MOF nucleation, (ii) attachment of nanoparticles onto MOFs through electrostatic interaction, and (iii) nanocomposite formation. SEM images of b) UiO‐66@NaYF_4_:Yb/Er, c) MOF‐801@NaYF_4_:Yb/Er, d,e) PCN‐223@NaYF_4_:Yb/Er, f) UiO‐66‐NH_2_@LiYF_4_, and g) UiO‐66‐NH_2_@NaGdF_4_ nanocomposites. Reproduced with permission.^[^ ^44^ ^]^ Copyright 2018, American Chemical Society. h) Photodynamic effects of the self‐assembled core‐satellite MOF‐UCNP superstructures (left) and random mixtures of MOFs and UCNPs (right). Reproduced with permission.^[^ ^46^ ^]^ Copyright 2017, Wiley‐VCH.

Surface ligand‐mediated synthesis requires complicated pre‐modification of the NPs. Recently, Huang et al. proposed the fabrication of UCNP‐MOF heterostructures through in situ self‐assembly approach driven by electrostatic interactions without the assistance of any capping agents (**Figure** **3**a).^[^ ^44^ ^]^ Briefly, the as‐synthesized, ligand‐free UCNPs were mixed with the MOF precursors (UiO‐66‐NH_2_ as the host material) in DMF and reacted at 120 °C for 24 h, yielding precipitated UCNP‐MOF nanocomposites. SEM and TEM images showed that UCNP‐MOF heterostructures were ~400 nm in size and exhibited octahedral geometry with UCNPs decorated on the MOF surface homogeneously, while no UCNPs were found in the inner part. Studies showed that, as the reaction proceeded, MOF nucleation formed small MOF crystals, accompanied by UCNPs initially attached to the MOF surfaces. With the continuous growth of MOF crystals, more UCNPs gradually decorated over the surface of MOF crystals just like cobblestones paving streets, finally UCNPs firmly and uniformly paved the surface of MOFs. In addition, the generality of this strategy has been proved by combining different kinds of MOFs (UiO‐66, MOF‐801, and PCN‐223) with UCNPs (LiYF_4_ and NaGdF_4_) to fabricate UCNP‐MOF nanocomposites (Figure 3b–g). These results suggested that UCNPs could decorate negatively charged MOF crystals and the formation of UCNP‐MOF heterostructures was neither affected by the type and morphology of MOFs nor by the composition of UCNPs. Such method could be further extended to design and fabricate more complicated, multifunctional nanocomposites, for example, a sandwiched MOF@UCNP@MOF nanostructure could be prepared by epitaxial growth of MOFs outer layer. Subsequently, Tian group reported the design and synthesis of core‐satellite superstructures composed of MOFs and UCNPs via similar self‐assembly strategy.^[^ ^45^ ^]^ The positively charged MOFs (UiO‐68‐NH_2_) were synthesized and loaded with photosensitizers (PSs) chlorin e6 (Ce6), and rose bengal (RB) to obtain the negatively charged MOFs, which further interacted with positively charged UCNPs to form hybrid nanostructures. Such post decoration approach would not affect PSs loading inside MOFs, and could ensure sufficient energy transfer efficiency from UCNPs to PSs.

### 2.3 DNA‐Directed Assembly

Due to its intrinsic merits of design flexibility and programmability, DNA has been considered as a powerful tool to assemble nanostructures with unparalleled precision and programmability.^[^ ^47^, ^48^ ^]^ DNA‐driven self‐assembly of nanomaterials has displayed site‐specific addressability and well‐ordered nanostructures, thanks to the nature of Watson‐Crick base pairing.^[^ ^49^, ^50^ ^]^ For example, Cha et al. prepared well‐defined, core‐satellite MOF‐UCNP heterostructures by DNA‐templated assembly of PCN‐224 MOFs and UCNPs (Figure 3h).^[^ ^46^ ^]^ During the synthesis, two complementary azide‐terminated DNA strands were conjugated on the surface of amine‐modified MOFs and UCNPs through click reaction to obtain DNA1‐MOFs and DNA2‐UCNPs, respectively. Then, core‐satellite MOF‐UCNP hybrid structures were constructed by annealing DNA1‐MOFs with DNA2‐UCNPs at 60 °C for 10 min. In addition, the assemblies can be precisely controlled by varying the molar ratios of MOFs to UCNPs. In contrast, only randomly‐distributed NPs were obtained by mixing UCNPs and MOFs modified with noncomplementary DNAs. Aptamers are short, single‐stranded functional oligonucleotides that can specifically bind to target molecules.^[^ ^51^ ^]^ Aptamer‐mediated formation of hybrid heterostructures offer potential opportunities for engineering DNA‐based nanodevices.^[^ ^52^, ^53^, ^54^ ^]^ Cheng and co‐workers synthesized UCNP‐MOF structures using aptamer modified UCNPs and MOFs, which could be used for the detection of bisphenol A in high‐salt foods.^[^ ^55^ ^]^ Aside from DNA base pairing interactions, *π*–*π* stacking interactions were adopted to adsorb aptamer‐modified UCNPs onto the surface of MIL‐101 to construct nanosensors for specific response to T‐2 toxin.^[^ ^41^ ^]^

## 3 Biomedical Applications of UCNP‐MOF Heterostructures

### 3.1 Bioimaging and Biosensing

Bioimaging represents a crucial tool for investigation of biological processes and cell metabolism, diagnosis of diseases, and monitoring therapy progression.^[^ ^56^, ^57^ ^]^ Compared with single modal imaging, multimodal bioimaging can provide more comprehensive information of diseases through multiple signals output, thus avoiding the inaccurate imaging results caused by the limitation of single‐modal imaging. UCNPs have been recognized as a promising candidate for bioimaging because of relatively high NIR light absorption capacity, which allows for deep tissue penetration, negligible photodamage, and auto‐fluorescence.^[^ ^58^ ^]^ MOFs can not only load adequate imaging agents benefiting from their structural flexibility and porosity, but also directly function as imaging agents.^[^ ^59^ ^]^ Notably, UCNP‐MOF heterostructures, which integrate the advantages of both MOFs and UCNPs and even endow with new properties, have demonstrated appealing applications in the field of multimodal bioimaging.

**Figure 4:**
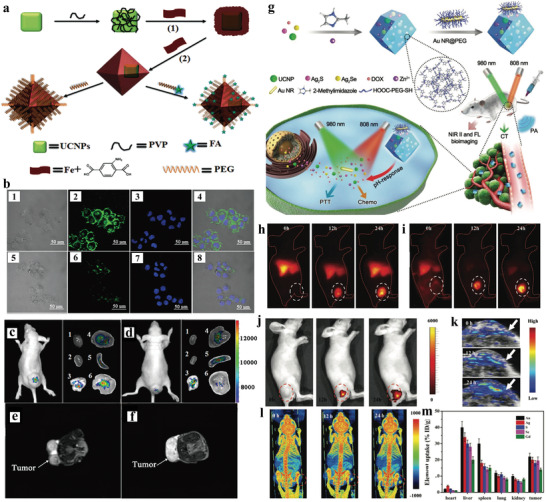
a) Schematic illustration of the synthesis of the core‐shell UCNP@Fe‐MIL‐101_NH_2_ nanostructures. b) CLSM images of the 1–4) KB cells and 5–8) MCF‐7 cells incubated with UMP‐FAs; UCL imaging of subcutaneous KB tumor‐bearing mice and dissected organs of the mice after intravenous injection of c) UMP‐FAs and d) UMPs. 1) heart; 2) kidney; 3) lung; 4) liver; 5) spleen; 6) KB tumor. The *T*_2_‐MRI images of dissected tumor of KB tumor‐bearing mice treated with e) UMP‐FAs and f) UMPs. Reproduced with permission.^[^ ^60^ ^]^ Copyright 2015, Wiley‐VCH. g) Schematic illustration of the synthesis of NPs@ZIF‐8@Au NR‐DOX for multimodal imaging‐guided combination phototherapy. In vivo FL imaging of h) Ag_2_S, i) Ag_2_Se, and j) UCL. k) PA and l) CT imaging of tumor‐bearing mice at different time points. m) Amounts of various elements in tissue samples measured by ICP‐MS. Reproduced with permission.^[^ ^61^ ^]^ Copyright 2018, Wiley‐VCH.

Tang group fabricated an octahedral core‐shell UCNP‐MOF hybrid nanocomposite (NaYF_4_:Yb,Er UCNP@Fe‐MIL‐101_NH_2_ (UMPs)) via surface ligand mediated growth for dual‐modal imaging of tumors (**Figure** **4**a).^[^ ^60^ ^]^ UCNPs could be used for fluorescence imaging due to upconversion luminescence (UCL) upon NIR excitation, while MOFs could be used for *T*_2_‐weighted magnetic resonance imaging (MRI). By integrating them into one heterostructure, the nanohybrids exhibited UCL/*T*_2_‐MR dual‐mode imaging properties. The UMPs were further modified with folic‐acid‐modified PEG (FA‐PEG) to obtain UMP‐FAs with improved biostability and tumor targeting capability. Under 980 nm laser excitation, human epidermoid carcinoma cells (KB cells, overexpressing the folate receptor (FR)) showed bright green UCL after 2 h UMP‐FAs treatment, whereas human breast cancer cells (MCF‐7 cells, FR‐negative) exhibited weak UCL, indicating that UMP‐FAs selectively target KB cells due to the specific recognition (Figure 4b). *T*_2_‐weighted MRI signals also suggested higher cellular uptake of UMP‐FAs by KB cells. In vivo studies revealed that mice bearing KB tumor showed intense UCL signals at the tumor site after 24 h of i.v. injection of UMP‐FAs, whereas much weaker signal was observed for the mice treated with UMPs (Figure 4c,d). Furthermore, the *T*_2_‐weighted MRI signal in tumor of mice treated with UMP‐FAs displayed a 35% decrease comparing to that treated with UMPs (Figure 4e,f). All these results confirmed the feasibility of the heterostructures for dual‐mode UCL/MR imaging.

Photoacoustic (PA) imaging is a novel noninvasive technique offering improved imaging depth with high spatial resolution.^[^ ^62^ ^]^ Recently, Kuang et al. presented that multimodality imaging which combined computed tomography (CT), PA, and UCL imaging could be realized in one hetero‐nanostructure (Figure 4g).^[^ ^61^ ^]^ They designed and synthesized multifunctional heterodimers made of gold nanorods (Au NRs) and ZIF‐8 encapsulated with UCNPs, Ag_2_S, Ag_2_Se, and doxorubicin hydrochloride (DOX). The system not only enabled NIR, CT, and PA multimodal imaging, but also allowed imaging‐guided photothermal therapy (PTT) and chemotherapy. Multifunctional NPs and DOX could be released from the decomposed ZIF‐8 structure under acidic tumor microenvironment, while the Au NRs attached to the MOF surface via electrostatic interactions was ready for NIR light‐mediated PTT. In vitro studies showed that UCL imaging was feasible with the observation of sufficient fluorescence signal, and acceptable combined therapeutic effect was obtained under safe treatment of 0.3 W cm^−2^ power density. Moreover, in vivo multimodal imaging demonstrated that the system could specifically accumulate at the tumor site in HeLa‐tumor‐bearing mice after 24 h post injection and generated significant signals for multimodal imaging (Figure 4h–m). The tumor‐bearing mice treated with the heterodimers displayed good therapeutic effect of combined PTT and chemotherapy. Moreover, Yang et al. developed core‐shell UCNPs@MIL‐100(Fe) NPs by a facile one‐pot method.^[^ ^63^ ^]^ Such UCNP‐MOF heterostructures not only achieved UCL, CT, and *T*_1_/*T*_2_ MR multimodal imaging, but also realized imaging guided synergistic anti‐cancer therapy. Given these results, the hybrid nanostructures represent promising platforms that can efficiently integrate several functional components to achieve multimodal imaging and combined therapy.

**Figure 5:**
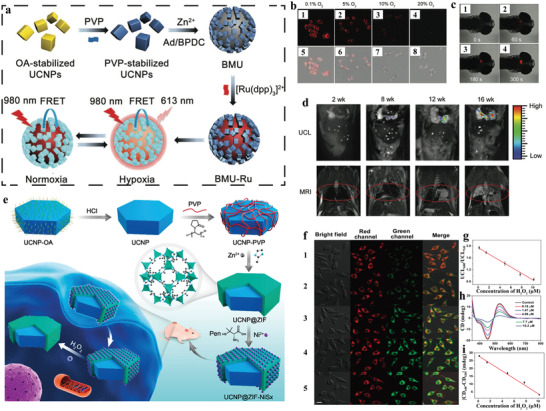
a) Schematic illustration of the synthesis of BMU‐Ru nanosensors for cycling hypoxia imaging. b) CLSM images of HeLa cells incubated with BMU‐Ru under various O_2_ concentrations. c) Zebrafish after treatment with BMU‐Ru for 1 h followed by addition of 15 mm BDM at different time. d) UCL and MRI images of the mice after intravenous injection of BMU‐Ru at different intervals (2, 8, 12, and 16 weeks). Reproduced with permission.^[^ ^65^ ^]^ Copyright 2020, Wiley‐VCH. e) Schematic illustration of the fabrication of UCNP@MOF‐NiSx nanoassemblies for ROS detection. f) Confocal images of the probe‐pretreated PCS‐460‐010 cells with addition of different concentrations of H_2_O_2_: 1) 0.18, 2) 1.47, 3) 4.89, 4) 7.7, and 5) 10.2 µM. g) Standard curves for H_2_O_2_ corresponding to fluorescence ratio (*I*_660_/*I*_540_). h,i) CD response of the HeLa cells pre‐incubated with the probe further treated with various concentrations of H_2_O_2_. Reproduced with permission.^[^ ^70^ ^]^ Copyright 2019, American Chemical Society.

In addition to bioimaging, detection of specific analytes in organisms reflected by the change of signal is indispensable for understanding biological processes, screening diseases, and revealing pathological mechanisms. On the basis of the optical properties of UCNPs^[^ ^64^ ^]^ and intrinsic biocompatibility and biodegradability of MOFs, UCNP‐MOF heterostructures offer unprecedented opportunities for biosensing. One widely adopted strategy is to construct FRET systems based on UCNP‐MOF heterostructures. For example, Tang et al. developed an NIR‐excited BMU‐Ru nanosensor for in vivo imaging of hypoxia (**Figure** **5**a).^[^ ^65^ ^]^ Hypoxia, an inadequate oxygen supply phenomenon, is one of the typical features of solid tumors.^[^ ^66^, ^67^ ^]^ The alterant hypoxia level effectively reflects the deterioration of the tumor, and is closely related to therapeutic effects.^[^ ^68^, ^69^ ^]^ In this study, the core‐satellite nanostructures were fabricated by attaching NaYF_4_:Yb,Tm@NaYF_4_ UCNPs onto the surfaces of bio‐MOF‐100, in which O_2_ quenchable indicator [Ru(dpp)_3_]^2+^Cl_2_ was encapsulated in the MOFs with a high loading content of 18.4 wt%. Under NIR excitation, FRET process occurred between UCNPs donors and [Ru(dpp)_3_]^2+^Cl_2_ acceptors, leading to bright red emission of [Ru(dpp)_3_]^2+^Cl_2_ peaked at 613 nm. The emission could be quenched by the increase of O_2_ concentration, thus allowing the monitoring of O_2_ concentration by BMU‐Ru nanosensors. The BMU‐Ru displayed improved sensitivity at low O_2_ concentration than free O_2_ indicator, which could be ascribed to the superior FRET efficiency (91.5%). The O_2_ sensing performance of the system was evaluated in several cell lines. Confocal laser scanning microscopy (CLSM) imaging demonstrated that the intense fluorescent signal at hypoxia condition (e.g., 0.1% O_2_) was remarkably suppressed when the O_2_ concentration gradually increased to 20% (normoxic condition) (Figure 5b). In addition, the signal response for O_2_ concentration was reversible for another three normoxia‐hypoxia cycles. The sensitivity and reversibility of the nanosensors were further confirmed in cerebral anoxia model of zebrafish (Figure 5c). Using genetically engineered murine model, non‐small cell lung cancer lesions could be tracked with clear and gradient UCL signals of BMU‐Ru nanosensors from 8 to 16 weeks during tumor progression (Figure 5d). Meanwhile, no apparent long‐term biotoxicity was observed 28 days post injection.

Reactive oxygen species (ROS), such as singlet oxygen (^1^O_2_), superoxide (O_2_^•−^), hydrogen peroxide (H_2_O_2_), and peroxyl radical (ROO·), are generated from the metabolism of oxygen and involved in many biological processes.^[^ ^71^ ^]^ Notably, excessive production of ROS would trigger oxidative damage to cell structures and biomolecules such as proteins and nucleic acids,^[^ ^72^ ^]^ thus have been implicated in various diseases including cancer, cardiovascular disease, Alzheimer's disease, and aging.^[^ ^73^ ^]^ Kuang and co‐workers developed a dual mode ROS biosensor (denoted UCNP@ZIF‐NiSx), which consisted of UCNP core and chiral NiSx NPs‐decorated ZIF shell, for chiral‐optical and fluorescent sensing of ROS (Figure 5e).^[^ ^70^ ^]^ The UCNP@ZIF‐NiSx presented two intense CD signals at 440 and 530 nm derived from chiral NiSx NPs and UCL signal from UCNPs that can be quenched by 535 nm absorption of NiSx NPs. The NiSx NPs could be decomposed by H_2_O_2,_ leading to the decrease of CD signal and the recovery of UCL at 540 nm while the UCL signal at 660 nm remained unchanged. Thus, the ROS level could be detected by the change of CD signal and the ratiometric UCL. The sensing performance was assessed in normal primary uterine fibroblast cells (PCS‐460‐010) (Figure 5f). Both the signal of CD and the ratiometric (*I*_600_/*I*_540_) signal showed a linear relationship with the intracellular H_2_O_2_ concentration ranging from 0.18 to 10.2 µM (Figure 5g–i). Moreover, the biosensor was successfully adopted to sense intracellular H_2_O_2_ level in HeLa cells and SK‐MEL‐2 cells with the results consistent with that using the H_2_O_2_ Kit. In vivo studies demonstrated that the system reflected rapid response to ROS. However, it is still inadequate for distinguishing recognition and quantitation of subtypes of ROS, similar to most of the existing ROS probes.

Nucleic acids including DNA and RNA play decisive roles in creation, storage, transfer, and expression of genetic information.^[^ ^74^ ^]^ Therefore, precise visualization of nucleic acids in organisms, particularly disease‐related DNAs and RNAs,^[^ ^75^, ^76^ ^]^ provides rich information about their roles and functions in various physiological and pathological processes.^[^ ^77^ ^]^ Recently, Xie et al. fabricated DNA‐hybrid‐gated UCNPs@MOF/DOX nanocomposites for fluorescent detection of miRNA‐21 (one of the oncogenic miRNAs overexpressed in various tumor cells) and on‐demand drug delivery.^[^ ^78^ ^]^ The 475 nm emission of UCNPs upon NIR irradiation could be suppressed by DOX loaded in the pores of MOF shells due to efficient FRET. In the presence of target, miR‐21 mediated toehold strand displacement reaction, leading to DNA gate collapse and release of DOX. DOX release was accompanied by the recovery of 475 nm emission of UCNPs due to the weakening of FRET, allowing for fluorescent detection of miR‐21 with a range of 4 to 500 nM. AS1411 modified nanoprobe could specifically recognize nucleolin on the cell membrane, thus the MCF‐7 cells displayed brighter UCL signal and more dead cells than that of normal HEK‐293T cells due to overexpressed miR‐21 and nucleolin in tumor cells. These illustrated that such hybrid biosensor was feasible for monitoring endogenous miR‐21 and triggering targeted drug delivery.

### 3.2 Drug Delivery

Currently, chemotherapy remains preferred clinical anticancer approach for cancer treatment.^[^ ^79^, ^80^ ^]^ The inherent limitations of chemotherapy such as toxic adverse effects, poor drug selectivity, have restricted its potential applications.^[^ ^81^, ^82^, ^83^ ^]^ With its porosity, large surface area, adjustable structure, and biodegradability, MOFs are attractive drug carriers that have been extensively explored.^[^ ^84^ ^]^ Additionally, the integration of luminescence into MOF‐based delivery systems would offer potential for optical tracking and monitoring of the drug delivery, thus is beneficial for improvement of therapeutic efficiency. Among them, UCNPs can harvest deep tissue‐penetration NIR light with excellent photochemical properties, which confer them with prominent advantages over other traditional fluorescent nanomaterials.^[^ ^85^ ^]^ It is no doubt that UCNP‐MOF heterostructures can be engaged in construction of novel drug delivery systems.

**Figure 6:**
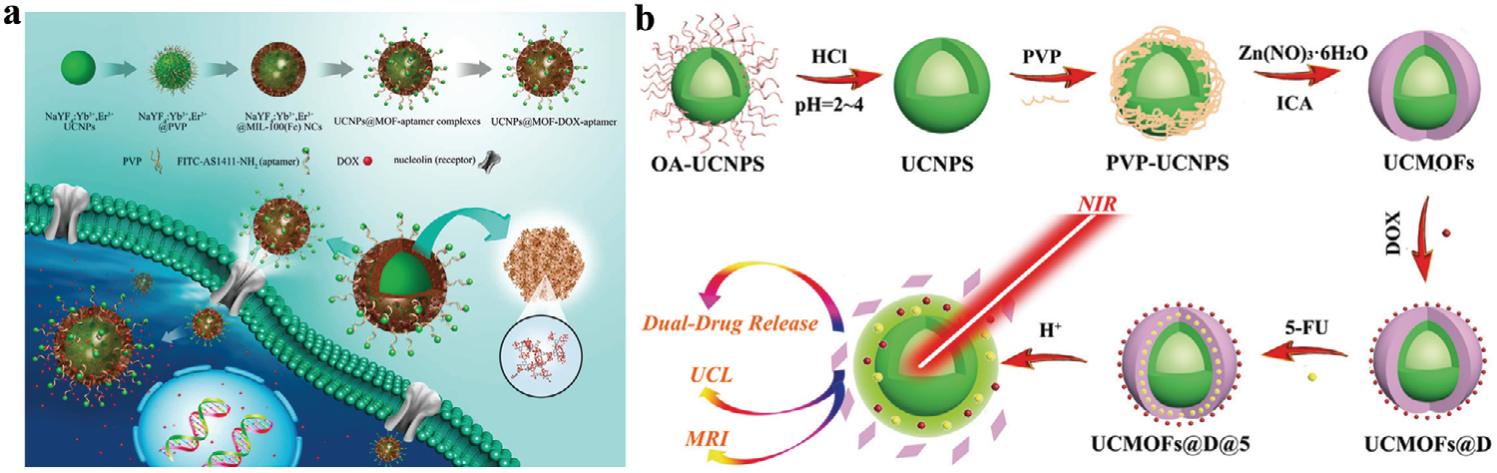
a) Schematic illustration of the synthesis of the aptamer‐modified NaYF_4_:Yb^3+^/Er^3+^@MIL‐100 for targeted drug delivery. Reproduced with permission.^[^ ^86^ ^]^ Copyright 2015, Springer Nature. b) Schematic of the UCMOFs for pH‐response drug release, UCL imaging and MRI. Reproduced with permission.^[^ ^87^ ^]^ Copyright 2020, The Royal Society of Chemistry.

Lin group reported the aptamer‐guided nanocarrier based on the core‐shell NaYF_4_:Yb^3+^/Er^3+^@MIL‐100(Fe) nanocomposites for targeted drug delivery and imaging (**Figure** **6**a).^[^ ^86^ ^]^ The UCNPs were used as optical labels for bioimaging under 980 nm laser, while MIL‐100 functioned as nanocarriers for DOX loading. The AS1411 aptamer was covalently conjugated on the surface of hybrid structures to specifically recognize nucleolin overexpressed on the cancer cells, which could improve cell targeting via receptor‐mediated endocytosis pathway. The bright fluorescence signals of confocal images and obvious binding shift of flow signals showed that the system was sufficiently internalized into MCF‐7 cells with high specificity but presented weak affinity to normal 293 cells. Eventually, the loaded DOX was released into MCF‐7 cells to specifically kill tumor cells with UCL monitoring.

To improve therapeutic efficiency and avoid systemic toxicity, tumor microenvironment‐responsive delivery systems have been developed for triggering drugs release at the targeted site. Among them, the low pH, a typical characteristic of tumor, is commonly selected as stimulus.^[^ ^88^, ^89^ ^]^ Several acidic pH‐responsive UCNP‐MOF heterostructures have been fabricated for targeted drug delivery, since MOFs could be degraded in acidic condition. For example, FA‐decorated UCNP@MOF heterostructures including UCNP@ZIF‐8,^[^ ^90^ ^]^ UCNP@UIO‐66(NH_2_),^[^ ^91^ ^]^ and UCNP@MIL‐53^[^ ^92^ ^]^ were developed for targeted delivery of anticancer drug DOX or 5‐fluorouracil (5‐FU). Integration of targeting function onto the heterostructures by surface modification of affinity ligands, such as aptamers and FA, could enhance the accumulation of nanocarriers in tumor cells by selective ligand‐receptor interactions. Combined with the pH‐sensitive degradation of MOFs, such hybrid nanocomposites achieved improved drug delivery efficiency. Moreover, nanocomposites doped by the metal ions such as Gd and Fe make it possible for tracking drug delivery with UCL/MR.^[^ ^93^, ^94^ ^]^

Single‐mode treatment is prone to cause undesirable side effects due to drug resistant, instability, and rapid elimination. Design of combinational therapy systems, such as co‐delivery of dual drugs, is a reasonable way to combat these limitations. Notably, the formulation of co‐delivery system requires more elaborate design as it should consider the corresponding release kinetics of distinct drugs. UCNP‐MOF heterostructures provide a reliable platform for construction of multi‐mode treatment system as demonstrated by Sun group. They proposed a theranostic nanoplatform (UCMOFs@D@5) for pH‐responsive delivery of dual drugs (DOX and 5‐FU) (Figure 6b).^[^ ^87^ ^]^ In this system, DOX and 5‐FU were encapsulated step‐by‐step into UCNP@MOF nanocomposites (UCMOFs) via Schiff base reaction and electrostatic interaction, respectively. In vitro drug release assays showed that DOX and 5‐FU were released from nanocarriers more quickly at low pH than neutral conditions, due to the disruption of covalent C–N bonds and the destruction of pH‐sensitive MOF structure under acidic microenvironment. It revealed that the UCMOFs@D@5 presented the superior efficacy of cells killing in contrast to single drug delivery system, thanks to the synergistic effects of dual drugs. Upon 980 nm excitation, the nanocomposites displayed bright intracellular fluorescence signal with high‐contrast, thus could be considered as a candidate for visual tracking of drugs delivery.

### 3.3 Photodynamic Therapy

PDT is an effective cancer treatment strategy with high selectivity and minimal damage to normal tissues.^[^ ^95^ ^]^ PDT relies on tumor localization of a PS to generate highly cytotoxic ROS under light irradiation to cause toxicity to cancer cells.^[^ ^96^ ^]^ During PDT process, the PS is first activated to the singlet excited state and subsequently enters to the triplet excited state (T1) via intersystem crossing process. The PS in T1 can transfer an electron to the surrounding substrate via hypoxia‐tolerant type I process to generate O_2_^•−^ or hydroxyl radical (·OH), or directly react with molecular oxygen (^3^O_2_) relying on oxygen‐dependent type II process to produce singlet ^1^O_2_.^[^ ^97^ ^]^ Therefore, therapeutic effect of PDT is mainly associated with PSs, light, and tissue oxygen. Without doubt, MOFs represent a promising delivery platform for PSs due to their tunable structures and high porosity. For example, PSs loaded in porous MOF are well‐separated due to its periodic structure, thus avoid aggregation and self‐quenching effect; the porous structure of MOFs allows ROS diffuse out easily to kill cancer cells. Besides, several MOFs are recognized as promising nanophotosensitizers (nPSs), such as DBP‐Hf, Hf‐TCPP, and PCN‐224, which could ensure higher ROS generation efficiency than molecular PSs because of the structural advantages and high accumulation in tumor sites due to enhanced permeation and retention effect. Such MOF‐based nPSs have been exploited as effective PDT agents for cancer therapy.^[^ ^98^ ^]^ In regard of the excitation wavelength, most of the PSs are stimulated by the relatively short wavelength with the limited light penetration depth, which restrict their further applications. UCNPs, as light transducers, can absorb NIR light and convert it into high photon energy, short‐wavelength emissions and activate PSs in deep tissues.^[^ ^99^, ^100^ ^]^ Due to the superior spectral overlap between the UCL emission of UCNPs and the absorption spectra of such MOFs, the energy transfer process could occur from the UCNP to the MOFs in UCNP‐MOF heterostructures, which confer such structures with NIR light‐harvesting capability for PDT. Moreover, precise regulation of heterostructural architecture would conduce to improve the energy transfer efficiency, thereby greatly ameliorate the efficacy of PDT. One would envision that UCNP‐MOF heterostructures could display unique advantages in PDT applications, including: 1) deep penetration depth with NIR exposure; 2) high ROS generation efficiency; 3) other unprecedented opportunities for combined therapy and imaging. Some accomplishments of using UCNP‐MOF heterostructures for PDT have been summarized in **Table** **1** and will be discussed in detail in the following paragraph.

**Table 1:** Summary of reported UCNP‐MOF heterostructures for PDT

UNCP‐MOF Heterostructures	Architecture	Additional functional materials	Therapeutic modalities	Ref.
(NaFY_4_:Yb/Er)‐(PCN‐224)	Core‐satellite	–	PDT	^[^ ^46^ ^]^
(NaGdF_4_:Yb,Tm@NaGdF_4_)‐(ZIF‐8)	Polyhedron	*g*‐C_3_N_4_, CDs	PDT	^[^ ^101^ ^]^
(UiO‐68‐NH_2_)‐(NaGdF_4_:Yb, Er@NaGdF_4_:Nd,Yb)	Core‐satellite	Ce6, RB	PDT	^[^ ^45^ ^]^
(NaGdF_4_:Yb,Er@NaGdF_4_)‐(PCN‐224)	Dimer	DOX	PDT + chemotherapy	^[^ ^37^ ^]^
(NaYF_4_:Yb/Er)@(ZIF‐8)	Core‐shell	MB, catalase	PDT	^[^ ^102^ ^]^
(MIL‐100(Fe))‐(NaYF_4_:Yb,Tm)	Core‐satellite	Fe_3_O_4_ NPs	PDT + PCT	^[^ ^103^ ^]^
(NaYF_4_:Yb/Er@NaYbF_4_:Nd@NaGdF_4_)@mSiO_2_@(ZIF‐90)	Core‐shell	DOX, RB, PEGFA	PDT + chemotherapy	^[^ ^104^ ^]^
(NaYF_4_)@(PCN‐224)	Dimer	TiO_2_	Type I and Type II PDT	^[^ ^105^ ^]^
(NaGdF_4_:Yb,Er@NaGdF_4_:Yb,Nd@NaGdF_4_)‐(PCN‐224)	Dimer	TPP	PDT	^[^ ^106^ ^]^
(NaYF_4_@NaYbF_4_:Er@NaYF_4_)@(PCN‐222)	Core‐shell	AuNPs	PDT + cancer starvation therapy	^[^ ^107^ ^]^
(NaGdF_4_:Yb,Er@NaGdF_4_)@(PCN‐224)	Core‐shell	TPZ, *α*‐PD‐L1	PDT + chemotherapy + immunotherapy	^[^ ^40^ ^]^
(NaYF_4_:Yb,Tm@NaYF_4_:Yb@NaNdF_4_)‐(PCN‐224(Fe))	Dimer	Biotin	PDT + CDT + SDT	^[^ ^108^ ^]^
(NaYF_4_:Yb,Er)‐(PCN‐224)	–	LA	PDT + gas therapy	^[^ ^109^ ^]^

#### 3.3.1 Single Photodynamic Therapy

Cha group constructed core‐satellite MOF‐UCNP heterostructures via DNA‐mediated assembly of UCNPs onto porphyrinic MOF (PCN‐224) surfaces.^[^ ^46^ ^]^ Such heterostructures exhibited improved PDT than simply mixing UCNPs and MOFs. Under 980 nm light irradiation, NaYF_4_:Yb/Er UCNPs emitted visible light to activate the porphyrins in PCN‐224 to generate ^1^O_2_. Cell viability test showed that MOF‐UCNP caused 63.7% cell death after 20 min irradiation, while only 48.1% cell death was observed when treated with the mixture of unassociated MOFs and UCNPs, demonstrating MOF‐UCNP superstructures could produce greater amount of ^1^O_2_ than that of generated by simply mixing UCNPs and MOFs. In addition, affibody‐modified MOF‐UCNP could specifically target MDA‐MB‐468 cells with overexpressed epidermal growth factor receptors with higher PDT efficacy.

**Figure 7:**
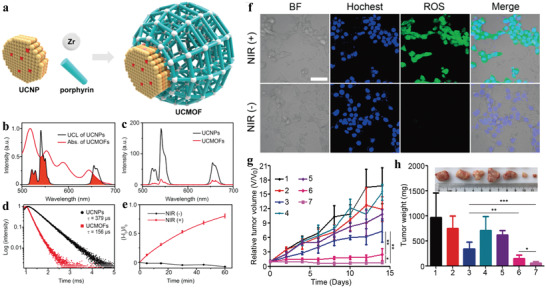
a) Schematic showing the photodynamic effect of the UCNP‐MOF heterodimers upon NIR light irradiation. b) UCL spectrum of the PVP‐coated UCNPs and UV–vis absorption spectrum of the UCMOFs. c) UCL spectra and d) UCL decay curves of the emission at 541 nm of PVP‐coated UCNPs and UCMOFs. e) ^1^O_2_ generation by UCMOFs with and without NIR irradiation, detected by SOSG assay. f) Confocal fluorescence images of 4T1 cells treated with UCMOFs and DCF‐DA, with or without NIR irradiation. Scale bar, 50 µm. g) The tumor growth curves after exposure to different treatments. h) Final weights of tumor tissues 14 days after treatment. Inset: representative images of the tumors for the seven groups of mice at day 14 (1: saline, 2: NIR, 3: DOX, 4: UCMOFs, 5: DOX/UCMOFs, 6: UCMOFs + NIR, 7: DOX/UCMOFs + NIR). Data are means ± SD; *N* = 5. *p < 0.05, **p < 0.01. Reproduced with permission.^[^ ^37^ ^]^ Copyright 2017, American Chemical Society.

For the heterostructures mentioned above, the distance between the energy donor (UCNP) and acceptor (MOF) was relatively long due to the DNA linker, which could reduce the energy transfer efficiency and compromise PDT efficacy. Recently, we reported the first NIR‐harvesting, asymmetric UCMOFs by selective anisotropic growth of PCN‐224 MOFs onto UCNPs and realized NIR light‐induced PDT (**Figure** **7**a).^[^ ^37^ ^]^ It is worthy to point out that the direct deposition of MOFs onto UCNPs lead to close proximity between the two nanodomains in the heterodimers. Such close proximity and spectral overlap (Figure 7b) could ensure the high energy transfer efficiency of more than 58% from UCNPs to porphyrins, as evidenced by time‐resolved photoluminescence measurements (Figure 7c,d). Under 980 nm irradiation, the system could generate ^1^O_2_ both in solution and in cells, while negligible ^1^O_2_ was observed without NIR irradiation as demonstrated by using SOSG assay in solution and dichlorofluorescein diacetate (DCF‐DA) as a cell permeable fluorescent probe (Figure 7e,f). More importantly, this strategy can be extended to construct heterodimers composed of core‐multi‐shell UCNPs and MOFs. For example, core‐four‐shelled UCNPs (NaGdF_4_:Yb,Er@NaYF_4_@NaYF_4_:Yb,Tm@NaYbF_4_:Nd@NaYF_4_) were synthesized and used for the construction of heterostructures with dual NIR light (980 and 808 nm) harvesting properties and tunable energy transfer from UCNPs to MOFs. Both in vitro and in vivo assays demonstrated that by loading DOX into the porous structure of the nMOF domain, the heterodimers presented enhanced potency for cancer treatment due to the combination of NIR‐activated PDT and chemotherapy (Figure 7g,h).

**Figure 8:**
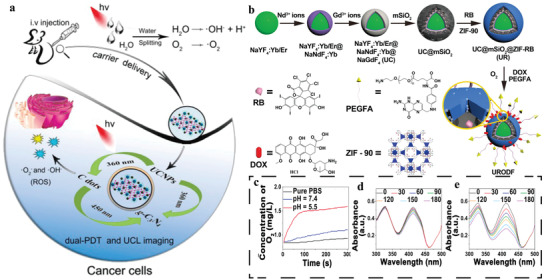
a) Schematic illustration of the mechanism of UCNPs‐*g*‐C_3_N_4_‐CDs@ZIF‐8 composite for PDT. Reproduced with permission.^[^ ^101^ ^]^ Copyright 2017, American Chemical Society. b) Schematic illustration of the preparation of URODF. c) O_2_ concentration of deoxygenated PBS solution treated with UC@mSiO_2_@ZIF‐O_2_ NPs. Black line in (c) was obtained from pure PBS solution (pH = 7.4) without other treatment. The absorbance changes of 1,3‐diphenylisobenzofuran treated with d) URDF and e) URODF in deoxygenated PBS buffer (pH = 5.5) after 808 nm laser irradiation for different time. Reproduced with permission.^[^ ^104^ ^]^ Copyright 2019, American Chemical Society.

To maximize utilization of light energy and further improve PDT efficiency, Yang et al. proposed a PDT system by integrating two PSs, carbon nitride (*g*‐C_3_N_4_) nanosheets and carbon dots (CDs), in one UCNP‐MOF hybrid structure (**Figure** **8**a).^[^ ^101^ ^]^ Under NIR irradiation, the NaGdF_4_:Yb,Tm@NaGdF_4_ UCNPs emitted UV light to activate *g*‐C_3_N_4_ to produce ROS. In addition, UCL could activate CDs to emit visible light that further reactivated *g*‐C_3_N_4_ to generate more ROS through stepwise water splitting. With ZIF‐8 shell protection, the nanocomposites were capable of generating sufficient ROS in buffer and at cellular level, which give rise to improve antitumor efficiency both in vitro and in vivo. Similar strategy was adopted by Tian group by fabrication of MOF@UCNP superstructures (CR@MUP) co‐loaded with two PSs, Ce6 and RB, whose absorptions were well overlapped with the emission of UCNPs.^[^ ^45^ ^]^ It validated that the dual PSs encapsulated nanostructures not only exhibited enhanced PDT effect and tumor suppression efficacy than the single‐PS system but also possessed trimodal (MR/UCL/FL) imaging functions.

As has been discussed, oxygen levels also affected PDT efficiency. Hypoxia, including PDT‐induced hypoxia or hypoxic tumor microenvironment, will largely compromise the therapeutic effect of PDT, which mainly relies on oxygen consumption type II mechanism.^[^ ^110^ ^]^ Notably, elevating oxygen supply allows to alleviate tumor hypoxia and ameliorate hypoxia‐induced the adverse impact of PDT treatment.^[^ ^111^ ^]^ There are several strategies to replenish oxygen content. One is delivery of O_2_ or nanomaterials capable of O_2_‐production to tumor sites.^[^ ^112^ ^]^ For example, Lin et al. reported the delivery of O_2_ using ZIF‐90 as an O_2_ reservoir to combat hypoxia (Figure 8b).^[^ ^104^ ^]^ They constructed ZIF‐90‐coated, RB‐loaded core‐shell UCNP‐mesoporous silica NPs (UC@mSiO_2_‐RB@ZIF) as PDT agents. Under acidic tumor microenvironment, ZIF‐90 shell could disassemble and quickly release O_2_ to enhance PDT in hypoxia conditions. As indicated in Figure 8c, ZIF‐90 underwent decomposition at pH 5.5 in PBS solution and the dissolved O_2_ concentration increased sharply, while similar response did not occur at pH = 7.4 or in other control group. Upon exposure to 808 nm laser, O_2_ release induced ROS enhancement was detected both in extracellular and intracellular environments (Figure 8d,e). In vitro cytotoxicity and in vivo anticancer treatment demonstrated that O_2_‐loaded system possessed effective tumor cell killing and higher tumor suppression effect than the group treated with PDT agents without O_2_ loading, which should be ascribed to stronger lethality of O_2_ supplement‐induced PDT enhancement. In addition, by further modifying tumor‐targeting molecule and DOX on ZIF‐90 shell (URODF), excellent anticancer therapeutic effect was achieved both in vitro and in vivo.

**Figure 9:**
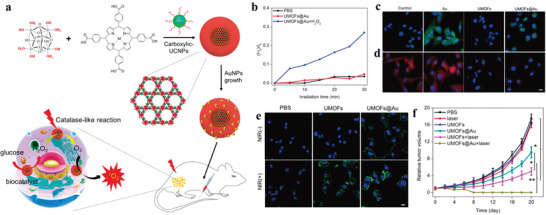
a) Schematic illustration of the synthesis of the core‐shell UMOFs@Au for synergistic cancer therapy via cascade catalytic reactions. b) ^1^O_2_ generation under normoxic conditions upon 980 nm laser irradiation. c) CLSM images of intracellular H_2_O_2_ detection. d) CLSM images of intracellular O_2_ detection under hypoxic conditions. Scale bar, 10 µm. e) CLSM images of apoptotic cells via Annexin V‐FITC assay (blue, DAPI; green, Annexin V). Scale bar, 10 µm. f) Tumor growth curves of mice bearing U87MG tumors subjected to various treatments. Data are shown as means ± SD; *N* = 5. Reproduced with permission.^[^ ^107^ ^]^ Copyright 2020, American Chemical Society.

Another way to elevate oxygen level is to convert other endogenous molecules, for example, H_2_O_2_, to O_2_ by biocatalysts. Hence, incorporation of biocatalysts into PDT agents represents a useful strategy to improve PDT efficiency. Furthermore, the consumption of H_2_O_2_ will also alleviate the malignancy of tumor, thus exerting a synergistic impact on PDT treatment.^[^ ^113^ ^]^ Tang group constructed a smart nanocomposite (UCNPs/MB@ZIF‐8@catalase) by attaching catalase onto the surface of core‐shell UCNPs/MB@ZIF‐8 to realize efficient PDT against hypoxic tumor.^[^ ^102^ ^]^ Methylene blue (MB) served as the PS and triggered the ^1^O_2_ generation upon UCL activation, while catalase catalyzed the conversion of endogenous H_2_O_2_ to O_2_ and further promoted ^1^O_2_ production. Nevertheless, the level of H_2_O_2_ in tumor cells does not have sufficient capacity to support abundant O_2_ production even catalase presents favorable catalytic performance. Rationally engineering tandem catalysis in PDT system would be an exciting and meaningful approach to overcome such barriers. Recently, Chen and co‐workers demonstrated the construction of MOF‐based biocatalysts/nanoreactors for cascade reaction driven PDT.^[^ ^107^ ^]^ The biocatalyst (UMOF@Au) was synthesized by the integration of ultrasmall AuNPs with core‐shell UCNP@MOF hybrid structures (**Figure** **9**a). In this cascade system, the ultrasmall AuNPs with glucose oxidase (GOx) like catalytic activity could effectively catalyze glucose depletion to generate a large amount of H_2_O_2_, giving rise to cancer starvation therapy. The generated H_2_O_2_ was then decomposed into O_2_ via the catalase‐like reaction catalyzed by the MOF shell to ensure continuous oxygen supply. Upon NIR irradiation, porphyrins in MOF shell were excited by the UCL emission of UCNPs to constantly produce cytotoxic ^1^O_2_ for PDT. Evaluation of ^1^O_2_ level in vitro by SOSG assay demonstrated that UMOF@Au exhibited similar outstanding ^1^O_2_ generation ability in normoxic conditions as that in O_2_‐free solutions with the addition of H_2_O_2_, while negligible ^1^O_2_ release was recorded under hypoxic conditions (Figure 9b). Moreover, the intracellular H_2_O_2_ and O_2_ concentration could also be increased by UMOFs@Au treatment, confirming the excellent catalytic performance of the UMOFs@Au NPs (Figure 9c,d). Upon NIR irradiation, cells treated with the system showed much higher PDT effect compared to cells treated with UMOF NPs and UMOF@Au NPs without laser irradiation, as evidenced by CLSM images of apoptotic cells (Figure 9e). Furthermore, the anticancer performance of UMOF@Au NPs was estimated in vivo and the system exhibited good tumor accumulation and excellent antitumor treatment effect, as proved by almost 100% tumor eradication on day 8 with no tumor recurrence (Figure 9f). The superior antitumor efficacy of UMOF@Au NPs was primarily benefited from the synergistic effect of cancer starvation therapy due to glucose depletion and NIR‐triggered, cascade catalysis reaction enhanced PDT.

Instead of increasing intratumoral O_2_ to relieve hypoxia, development of O_2_‐independent type I PDT is another choice to avoid the thorny problems caused by hypoxia.^[^ ^114^ ^]^ For example, titanium dioxide (TiO_2_) is a kind of photocatalyst that can be activated by UV light to generate ROS via type I process. Recently, Yang et al. fabricated a nanoplatform through combination of ultrasmall TiO_2_ NPs and UMOFs, realizing type I and type II multimode PDT with enhanced therapeutic efficacy.^[^ ^105^ ^]^ Confocal results showed that the MCF‐7 cells incubated with UMOF‐TiO_2_ displayed obvious red and green fluorescence signals originating from products of type I and type II PDT, while only green fluorescence signal of type II PDT product was observed for MCF‐7 cells treated with UMOF. The MTT assay manifested that UMOF‐TiO_2_ with multimode PDT abilities exhibited higher phototoxicity and killing potency toward tumor cells under NIR irradiation, compared with type II PDT‐treated cells. Subsequently, the photoinduced tumor ablation capacity of UMOF‐TiO_2_ was also verified by tissue staining and in vivo experiments.

**Figure 10:**
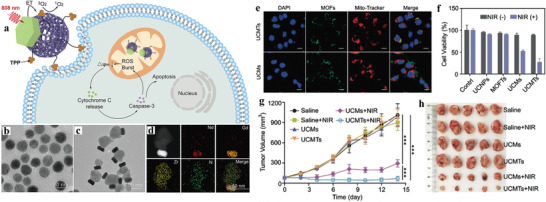
a) Schematic of 808 nm NIR light‐activated, mitochondria‐targeted UCMT for amplified PDT. TEM images of b) NaGdF_4_:Yb,Er@NaGdF_4_:Yb,Nd@NaGdF_4_ UCNPs and c) UCMTs. d) Elemental mapping images corresponding to HAADF‐STEM image of a single UCMT. e) CLSM images of the 4T1 cells treated with UCMTs or UCMs. f) Cell viability of 4T1 cells treated with different samples with or without 808 nm laser irradiation. g) The tumor growth curves after intratumoral injection of different samples followed with or without NIR irradiation. Data are means ± SD; *N* = 5. ***p < 0.001. h) The tumor tissues photographed after different treatments. Reproduced with permission.^[^ ^106^ ^]^ Copyright 2019, Wiley‐VCH.

Mitochondria, as cellular powerhouse and weapon store, plays crucial roles in a myriad of biological processes.^[^ ^115^, ^116^ ^]^ Mitochondria has been considered as a potential therapeutic target for cancer, since mitochondrial dysfunction would interrupt energy replenishment and activate mitochondria‐mediated cell death machinery.^[^ ^117^, ^118^ ^]^ Particularly, modulation of the ROS levels inside mitochondria for disruption of its homeostasis is now emerging as an attractive strategy to maximize PDT efficacy.^[^ ^119^ ^]^ However, the short lifetime of ROS (≈40 ns for ^1^O_2_) and limited diffusion range from its site of generation (about 20 nm) have hindered its migration to mitochondria and reduced mitochondria‐mediated therapeutic responses. Therefore, localization of PSs in specific subcellular organelles of targeted cells is conducive to ameliorate the therapeutic effect of PDT.^[^ ^120^ ^]^ To tackle such issue, we developed an NIR‐triggered UCNP‐MOF heterostructure (UCMT) with mitochondria‐targeting capability and achieved amplified PDT with high spatiotemporal precision (**Figure** **10**a).^[^ ^106^ ^]^ The UCMTs were synthesized via asymmetric modification of the mitochondria‐specific ligand triphenylphosphine (TPP) onto the MOF domain of the as‐prepared Janus NPs (UCM), which were made of Nd^3+^‐sensitized UCNPs and porphyrinic MOFs (Figure 10b–d). Colocalization experiments performed in 4T1 cells showed that fluorescence of UCMTs overlapped well with signals of Mito‐Tracker Red, in contrast, UCMs showed poor overlapping with mitochondria, verifying that TPP was indispensable for the accumulation of UCMTs into mitochondria (Figure 10e). Upon 808 nm light irradiation, UCMTs produced more intracellular ^1^O_2_ than that was produced by UCM and MOFT (TPP‐modified MOF NPs). Consistent with the ^1^O_2_ generation capacity, UCMTs also demonstrated excellent photodynamic cytotoxicity compared to UCM and MOFT, as evidenced by the standard cell viability assay (CCK‐8 assay) (Figure 10f). Subsequently, the anti‐cancer performance in vivo demonstrated that UCMTs inhibited tumor growth more effectively than UCM after the same NIR irradiation, further confirming that mitochondria‐targeting strategy offered a positive impact on improving the efficiency of PDT (Figure 10g,h). Moreover, the 808 nm light excitation also exerted minimum overheating effect and damage to normal tissue in comparison with 980 nm laser, which is more suitable for PDT.

#### 3.3.2 Combination Therapy

**Figure 11:**
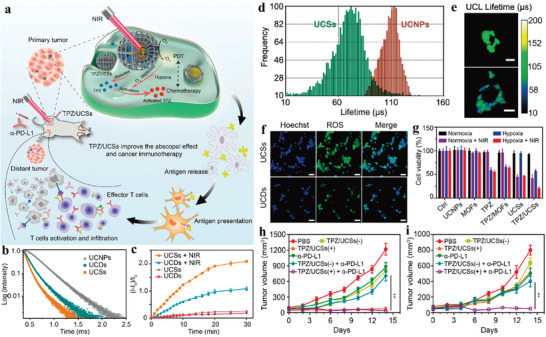
a) Schematic illustration of the structure of TPZ/UCSs and their application for tumor treatment through a combination of NIR light‐triggered PDT and hypoxia‐activated chemotherapy with immunotherapy. b) UCL decay curves of UCNPs, UCDs, and UCSs. c) NIR light‐induced ^1^O_2_ generation by UCDs or UCSs determined by SOSG assay. d) UCL lifetime distributions of UCNPs and UCSs in CT26 cells. e) Lifetime images of UCL at 542 nm in CT26 cells treated with UCNPs or UCSs. f) Fluorescent images of CT26 cells incubated with UCDs or UCSs, followed by NIR light irradiation. Scale bar, 50 µm. g) Cell viability of CT26 cells (cultured in a hypoxic (2%) or normoxia (21%) atmosphere) with different treatments. Growth curves of h) primary tumors and i) distant tumors in CT26 tumor‐bearing mice after different treatments. 1) PBS, 2) TPZ/UCSs(−), 3) TPZ/UCSs(+), 4) *α*‐PD‐L1, 5) TPZ/UCSs(−) + *α*‐PD‐L1, and 6) TPZ/UCSs(+) + *α*‐PD‐L1. Data are means ± SD; *N* = 5. ns: not significant. Reproduced with permission.^[^ ^40^ ^]^ Copyright 2020, American Chemical Society.

Given that the efficacy of PDT is largely suppressed by intrinsic biological responses and resistance, such as excretion of PSs by transporters or exocytosis and the activation of the antioxidant system, meanwhile such situation is difficult to ameliorate through modulation of the aforementioned strategies.^[^ ^112^ ^]^ Thus, the combination of PDT and other therapeutic modalities via different mechanisms in one platform can be harnessed to minimize the resistance to monotherapy.^[^ ^121^ ^]^ Previous pioneering work reported an NIR‐harvesting UCNP‐MOF heterodimer by PVP‐mediated synthetic strategy and realized an efficient combined cancer treatment via NIR‐induced PDT and DOX‐mediated chemotherapy. Although relatively satisfactory tumor curative effect was obtained, energy loss due to long distance between two modules of heterodimers and the adverse effects of chemotherapy on normal tissues could attenuate the combined therapeutic efficacy. To combat this, we engineered a core‐shell UCNP@MOF nanoplatform (UCSs) via citrate acid (CA)‐mediated MOF growth to combine NIR‐induced PDT with hypoxia‐activated chemotherapy to achieve improved cancer treatment against hypoxic tumors (**Figure** **11**a).^[^ ^40^ ^]^ Compared with asymmetric heterodimers (UCDs), symmetric UCSs exhibited enhanced FRET efficiency (Figure 11b–e) due to the shortened distance between UCNP core and MOF shell and higher enabled ^1^O_2_ generation efficiency upon NIR light triggering (Figure 11f). Tirapazamine (TPZ), a hypoxia‐activated prodrug, was encapsulated into the nanopores of the MOF shell to obtain final constructs (TPZ/UCSs). According to cell viability and cell apoptosis tests, TPZ/UCSs displayed specific killing ability against hypoxic cells. The CT26 cells incubated with TPZ/UCSs in hypoxia upon NIR irradiation led to greater cytotoxicity compared to that of the cells under the same treatment without NIR irradiation, which indicated that the integration of NIR light‐induced PDT and hypoxia‐activated chemotherapy could improve the cell killing efficacy (Figure 11g). Subsequently, in vivo assay has proved that TPZ/UCSs possessed good performance of antitumor efficacy. Further combination of TPZ/UCSs with checkpoint blockade immunotherapy agent (anti‐programmed death‐ligand 1, *α*‐PD‐L1) not only suppressed primary tumors completely but also inhibited the growth of untreated distant tumors by eliciting the abscopal effect (Figure 11h,i). According to the percentage of tumor‐infiltrating, the antitumor immunological mechanism of *α*‐PD‐L1 checkpoint‐blockade and TPZ/UCSs was attributed to the effectively increased proportion of infiltration T cells in both primary and distant tumor sites.

Moreover, Yang et al. proposed a synergistic PDT nanoplatform based on construction of PS‐loaded Fe_3_O_4_@MIL‐100(Fe) heterostructures.^[^ ^103^ ^]^ UCNPs were covalently attached onto MIL‐100(Fe) surfaces and functioned as light transducers. The UV–vis UCL could excite the PSs and promote generation of ·OH because heterojunctions greatly restricted the recombination of electrons and holes. Meanwhile, the NIR‐mediated photo Fenton reaction around MOF could produce more ·OH. Both in vitro and in vivo assays proved that the combination therapy possessed outstanding antitumor ability.

**Figure 12:**
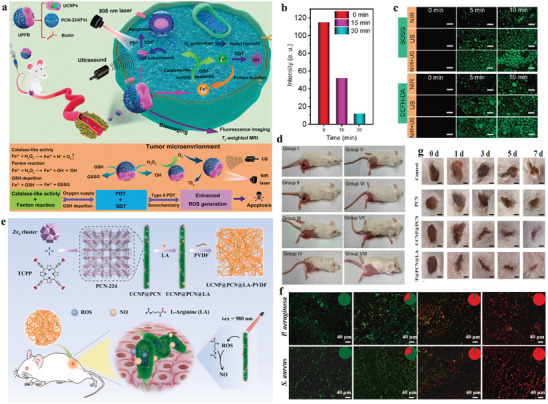
a) Schematic illustration of antitumor mechanism of UPFB. b) HeLa cells were stained with [Ru(dpp)_3_]Cl_2_ as dissolved O_2_ probes and then treated with UPFB at different incubating time. c) Detection of ROS generation of UPFB in vitro. The generation of ^1^O_2_ was confirmed by SOSG and the ·OH was detected by DCFH‐DA. d) The digital photos of mice after different treatments for 14 days. The groups were I) control, II) 808 nm laser, III) US, IV) UPFB + DMTU, V) UPFB + DMTU + NIR, VI) UPFB + DMTU + US, VII) UPFB + DMTU + NIR + US, and VIII) UPFB + NIR + US. Reproduced with permission.^[^ ^108^ ^]^ Copyright 2021, American Chemical Society. e) Schematic illustration of the preparation and bactericidal activity of UCNP@PCN@LA‐PVDF nanocomposites. f) CLSM images of *Pseudomonas aeruginosa* and *Staphylococcus aureus* attached on PET films treated by different membranes. g) Representative photographs of infected wounds after different treatments for 0, 1, 3, 5, and 7 days (scale bar is 2.5 mm). Reproduced with permission.^[^ ^109^ ^]^ Copyright 2020, Elsevier B.V.

Recently, Lin group designed and synthesized UCNP‐MOF Janus structures (UPFB) for synergistic tumor therapy including PDT, chemodynamic therapy (CDT), and sonodynamic therapy (SDT) (**Figure** **12**a).^[^ ^108^ ^]^ In this work, ultrasound was selected as an exogenous energy source for activation of UPFB to heighten ROS generation, which made up for the inefficient PDT caused by low energy transfer in the heterodimers. Besides, Fe^3+^ ions coordinated in porphyrin‐based PCN‐224(Fe) MOFs served as catalase‐like nanozymes, which not only catalyzed the decomposition of H_2_O_2_ to O_2_ to alleviate tumor hypoxia (Figure 12b), but also suppressed other pathways consuming generated ROS by intracellular GSH depletion. Meanwhile, Fe^2+^ generated during redox reaction of Fe^3+^ with H_2_O_2_ could further react with H_2_O_2_ as Fenton catalyst to yield toxic ·OH under acidic conditions, thus accomplishing CDT performance (Figure 12c). Additionally, the author prepared biotin functionalized system for specific recognition of overexpressed biotin receptors in tumor cells. The results showed that biotin functionalization would promote UPFB accumulation at the target site in comparison to UPF without biotin. UPFB displayed the synergistic antitumor effect in vivo (Figure 12d).

Apart from anti‐cancer therapy, bacteria‐killing is another major application of PDT,^[^ ^122^ ^]^ as ROS generated by PDT process can exert irreversible damage to bacterial cell membrane and the DNA inside. However, antibacterial performances are also compromised by the natural limitation of ROS.^[^ ^123^ ^]^ Notably, nitric oxide (NO), emerging as a type of gas therapy, has widely participated in antibacterial applications with broad‐spectrum antibacterial activities.^[^ ^124^ ^]^ Benefiting from long half‐life and wide diffusion radius, NO possesses a larger sterilization area than ROS, which can offset the lack of biocidal area of ROS.^[^ ^125^ ^]^ Zhao and co‐workers developed UCNP@PCN nanocomposite membrane for NIR triggered, NO‐assisted antibacterial therapy (Figure 12e).^[^ ^109^ ^]^ UCNP@PCN was first loaded L‐arginine (LA) and then incorporated into polyvinylidene fluoride (PVDF) matrix via an electrospinning approach to achieve UCNP@PCN@LA‐PVDF nanocomposite membrane. Upon NIR irradiation, UCNP@PCN could generate sufficient ROS to kill bacteria. The generated ROS also reacted with LA to produce NO, leading to NO‐assisted PDT antibacterial performance. In vitro antibacterial activity assay showed that the system could activate the sterilization with a larger range and superior bactericidal performances against Gram‐positive and Gram‐negative bacteria compared to the UCNP@PCN‐PVDF with only PDT effect, indicating that NO‐assisted PDT strategy could effectively facilitate the antibacterial efficacy (Figure 12f). Moreover, the studies on wound healing in mice further proved excellent synergistic antibacterial and anti‐inflammatory capabilities of the membrane (Figure 12g).

## 4 Conclusion and Perspectives

In the past few years, the rapid development of nanotechnology has boosted the design and synthesis of multifunctional UCNP‐MOF heterostructures. As have been summarized in this review, such UCNP‐MOF heterostructures have integrated the unique optical properties of UCNPs, such as tunable UCL, excellent photostability, and deep‐tissue‐penetrable light transducer with the advantageous characteristics of MOFs such as tailorable porosity, synthetic tenability, and structural regularity, thus enabling them ideal candidates for various applications in biosensing, imaging, drug delivery, and PDT. To date, UCNP‐MOF hybrid materials with variable structures have been fabricated through different strategies. These heterostructures not only preserve the respective advantages of each component, but also present unprecedented benefits over the single system by elaborated association, thus further promote their potential for bio‐applications. Despite of the remarkable progress made, more efforts should be devoted to overcome the key challenges included in the following:

i) Developing feasible and general strategies for controllable synthesis and assembly of UCNP‐MOF heterostructures remains a great challenge. Current methods mainly rely on the formation of MOF crystal structure on the surface of as‐prepared UCNPs. However, large lattice mismatch and the uncontrollability of the nucleation and growth rate of MOFs make it difficult to grow according to the programmable design. Meanwhile, the underlying mechanism for the formation of heterostructures is less explored due to the complexity of these systems. In fact, these seemingly inconsistent results have been obtained when researchers tried to investigate the role of ligands for the synthesis of asymmetric UCNP‐MOF heterostructures. The role of different types of MOFs or UCNPs, experimental conditions and surface ligands in the formation of heterostructures are still ambiguous and need to be investigated systemically.
ii) The utilization of UCNP‐MOF nanocomposites as drug delivery vehicles currently relies on the porous structures of MOFs for incorporation of drugs and drug release kinetics mainly depends on the MOF degradation. Thus, designing controlled drug release systems based on UCNP‐MOF heterostructures, for example, by capping the MOF pores with stimuli‐responsive ligands and using specific triggers to remove the capping ligands, may spark new inspiration on the application of UCNP‐MOF heterostructures. Recently, an amino acid‐boosted biomimetic strategy has been reported that enabled rapidly encapsulate or coencapsulate a broad range of proteins and enzymes into MOFs. Such strategy could keep the native conformations and stability of encapsulated proteins or enzymes,^[^ ^126^ ^]^ which may allow constructing UCNP‐MOF heterostructure to realize on demand delivery of biomacromolecules.
iii) In regard of biosensing and PDT, a major concern is that most of the reported UCNPs exhibit relatively low quantum efficiency of UCL due to the surface quenching effect and the energy loss during energy transfer process, which have a negative impact on the imaging performance of heterostructures and greatly compromise the energy utilization efficiency thereby limit the PDT efficacy, particularly for treatment of deep‐sited tumors. Thus, detailed understanding of the influence of each component, including the host, dopant, and surface deactivations on the upconversion efficiency can guide the precise design and regulation of UCNP structures and compositions, which eventually help to develop novel UCNP‐based heterostructures. Although different strategies have been proposed for UCL enhancement, further improvement is urgently needed.
iv) Although the heterostructures bring more possibilities for all‐in‐one combined treatment, more efforts are needed to explore the optimal combinations of different therapeutic modalities in order to obtain potent efficacy and minimum systemic toxicity before being applied in clinical settings. This may include the comprehensive consideration of biological reactions in accordance with their kinetics, sequential administration of various treatments, as well as coordinated utilization of external and internal stimuli.
v) Biosafety concern is the most fundamental issue for these heterostructures in clinical and pharmaceutical applications. While the short‐term stability and safety of UCNP‐MOF heterostructures in biological systems have been proved acceptable, the long‐term toxicological information and enrichment characteristics in large animals have not been explored but entail systematical and exhaustive investigation.
vi) The excitation wavelength of conventional UCNPs is concentrated in the NIR I window (980 nm or 808 nm), which overlaps well with the absorption range of water molecule. Recent years, NIR II window‐responsive imaging and therapy have attracted intensive interest due to reduced photon scattering, deeper tissue penetration, high‐resolution imaging, and lower absorption featured by NIR II spectral region (range from 1000 to 1700 nm) compared with NIR I. Therefore, developing NIR II‐excited UCNP‐MOF nanocomposites for precise imaging and potent therapy remains a key challenge for improved performance. Recently, Zhang et al. presented the design and synthesis of an energy‐migratory Yb sublattice in an Er‐sensitized nanostructure and achieved NIR II‐excited photon up‐conversion with large anti‐Stokes shift for a broad range of lanthanide ions.^[^ ^127^ ^]^ Such intriguing achievement will provide new opportunities for construction of NIR II‐responsive UCNP‐MOF heterostructures with new functions and markedly expand their clinical application potential.

## Conflict of Interest

The authors declare no conflict of interest.
